# Audit data governance for disability-inclusive public services: A systematic review and integrative S–A–C framework

**DOI:** 10.1371/journal.pone.0350135

**Published:** 2026-05-22

**Authors:** Sitthisak Chaiyasuk, Krish Rugchatjaroen, Somboon Sirisunhirun, Nopraenue Sajjarax Dhirathiti, Somsak Amornsiriphong, Phut Ploywan

**Affiliations:** 1 State Audit Office of the Kingdom of Thailand, Bangkok, Thailand; 2 Faculty of Social Sciences and Humanities, Mahidol University, Nakhon Pathom, Thailand; 3 International College, Mahidol University, Nakhon Pathom, Thailand; Queensland University of Technology, AUSTRALIA

## Abstract

This study consolidates interdisciplinary evidence on how audit data governance shapes accountability and service equity in data-intensive public services, with specific sensitivity to disability-inclusive delivery. Guided by the Preferred Reporting Items for Systematic Reviews and Meta-Analyses (PRISMA) 2020, we conducted a protocol-driven systematic literature review of peer-reviewed studies and selected institutional and standards sources published between 2010 and 2025. We searched Scopus and complemented database retrieval with targeted searches of audit, digital government, accessibility, and data-governance portals. We applied a theory-led narrative synthesis to accommodate heterogeneity in designs, domains, and outcome reporting. Synthesizing 125 records, we find that governance effects are produced through three interacting dimensions—service delivery for people with disabilities (S), audit data governance (A), and data-driven culture (C)—that jointly condition transparency, contestability, and distributive impacts. Service delivery arrangements shape where data are generated, which encounters are recorded, and whether access needs, reasonable accommodations, and complaints become organizationally legible. Audit data governance conditions the credibility and usability of audit trails through provenance, metadata, retention, audit logging, and role-appropriate oversight access, shaping both oversight feasibility and privacy risk. Data-driven culture conditions how evidence is interpreted and acted upon, including data literacy, the standing of experiential evidence, and whether audit signals support learning or only compliance. Across the corpus, recurring tensions emerge between optimization and inclusion; transparency and privacy; standardization and reasonable accommodation; automated decisioning and meaningful contestability; and compliance-oriented auditing and learning-oriented governance. Drawing these mechanisms together, we propose an integrative framework linking S, A, and C to explain how auditability and disability inclusion are co-produced across the digital service lifecycle. The framework identifies leverage points for governance reform and evaluation and outlines implications for future empirical testing and for practitioners seeking trustworthy, oversight-ready, inclusive digital public services. A review protocol was prepared before the review; the review was not registered.

## Introduction

Trust is a core currency of democratic governance. It conditions whether people perceive public institutions as legitimate, fair, and worth engaging with. For people with disabilities, trust is not formed only at the service counter or on a government website. It is also shaped upstream—by how public organizations collect, classify, govern, and interpret the information that structures eligibility decisions, service pathways, performance claims, and complaints. When information infrastructures fail to reflect disabled people’s everyday realities, the consequences are not merely administrative. They can translate into misrecognition, procedural friction, and unequal outcomes across both paper-based and digital services [1–3].

This raises a governance problem that sits squarely at the intersection of public policy and information technology: how should governments govern the data that underpin public accountability so that public services become more equitable—and more trustworthy—for people with disabilities? Digitalization has expanded what states can record and measure, but it has also amplified the consequences of omissions and misclassifications. As governments increasingly “govern by data,” decisions about categories, evidentiary thresholds, traceability, and authorized uses become consequential sites of power—shaping what is visible, actionable, and contestable in public administration [4]. In disability-related services, where administrative categories often mediate access to rights and support, these design choices can determine whether service users experience the state as enabling, indifferent, or exclusionary [1,5].

Accountability regimes intensify these dynamics. Modern public management has long relied on audit and performance systems to make administration legible and controllable, yet audit is not a neutral mirror of reality. It is an institutional practice that privileges certain forms of evidence, routinises what counts as “good performance,” and can crowd out experiential forms of knowing [6]. In digital government, this becomes increasingly data-specific: which datasets are treated as “audit-relevant,” how those data are generated through service processes, and how they are governed across organizational boundaries. Audit-relevant data in disability-related services include, for example, eligibility and verification records, categorization rules, case-management logs, digital identity checks, service transactions, performance indicators, and grievance or complaint pathways—each shaping what can be evidenced, questioned, and corrected. Yet the governance of these audit-relevant data is rarely theorized in a way that foregrounds disability equity and trust outcomes, even though such data increasingly shape what public organizations claim to have delivered, to whom, and with what justification.

A disability-equitable account of this problem cannot focus on service users alone. Public officials—both policy designers and street-level implementers—are pivotal actors in how audit-relevant data are produced and interpreted. Their incentives and interpretive frames are shaped by administrative paradigms and reform logics, including efficiency-oriented managerialism associated with New Public Management, value- and citizenship-oriented administration associated with New Public Service, and collaborative, networked service delivery associated with New Public Governance [7–9]. These paradigms carry different assumptions about what should be measured, what counts as “fair process,” and which values should dominate when trade-offs arise. A credible governance solution, therefore, must treat disability-inclusive accountability as a shared interpretive project—one that bridges experiential judgments of service adequacy and dignity with institutional judgments of compliance, performance, and legitimacy.

Existing frameworks provide partial guidance but remain fragmented. Public administration reform frameworks—such as the OECD/SIGMA Principles of Public Administration, developed by the Organisation for Economic Co-operation and Development (OECD) and the Support for Improvement in Governance and Management (SIGMA) programme and their assessment methodology—articulate standards for capable, accountable, and citizen-oriented administration, but they are not designed to specify how audit-relevant data should be governed in digital service environments [10]. In the audit domain, the International Organization of Supreme Audit Institutions (INTOSAI) emphasizes that Supreme Audit Institutions should “make a difference to the lives of citizens,” and the INTOSAI Development Initiative (IDI) guidance stresses strategic management and value creation; however, operational links between audit value, data governance arrangements, and disability-inclusive services remain under-specified [11,12]. In information governance, the Data Management Association (DAMA)’s Data Management Body of Knowledge (DMBoK) offers comprehensive data management guidance, while Transform Health’s Health Data Governance Principles foreground rights, equity, and accountability; yet these are rarely connected explicitly to audit systems and public trust in disability-relevant services [13,14]. Finally, the organizational capability to enact any governance design depends on culture: data-driven decision-making requires norms, routines, and leadership commitments that shape how data are valued, challenged, and used [15].

As a result, the evidence base is dispersed across digital government, public administration, audit studies, information governance, and disability research. Studies of trust often focus on adoption, satisfaction, or perceived risk in e-government, but rarely examine how the governance of audit-relevant data mediates trust formation over time [16,17]. Disability scholarship richly documents access barriers and the politics of classification, yet seldom connects these mechanisms to the auditability and accountability infrastructures that increasingly govern public services [1,3]. Meanwhile, technical and managerial work on data governance tends to privilege architecture and control, without systematically integrating disability equity and trust as core evaluative criteria [13,14]. Practitioners therefore lack an integrated conceptual map for designing governance arrangements that make disability-related public services simultaneously auditable, equitable, and trust-enhancing.

This article addresses that gap through a systematic literature review of a corpus exceeding one hundred studies, supplemented by structured engagement with four foundational frameworks: (i) OECD/SIGMA public administration principles and assessment methodology (OECD/SIGMA, 2019), (ii) SAI value and strategic management guidance [11,12], (iii) data governance and health data governance principles [13,14], and (iv) data-driven culture as an organizational enabler [15]. Because study designs and outcomes are heterogeneous, the review employs theory-led synthesis rather than statistical meta-analysis. Synthesizing these streams, we develop and label a concept that we term **audit data governance**: the institutional arrangements through which public organizations govern the data that render service performance and administrative decisions auditable, while ensuring that such data meaningfully represent disabled people’s service realities and can support equity- and rights-affirming accountability.

To explain how trust and equity outcomes are jointly shaped in disability-related public services, we organize the synthesis into three interlocking lenses: **service delivery for people with disabilities (S)**, **audit data governance (A)**, and **data-driven culture (C)**. We argue that design choices in service processes and information flows shape what becomes “audit-relevant” and how representationally valid those data are; governance arrangements determine whether data are secure, interpretable, traceable, and contestable across organizational boundaries; and organizational culture conditions whether governance is enacted as everyday practice rather than formal policy. The review makes three contributions. First, it consolidates a fragmented literature into a coherent conceptual landscape that centers disability equity and trust as evaluative anchors. Second, it proposes a framework explaining how **A can narrow interpretive gaps between service users and public officials without presuming divergent constructs across groups**: by establishing a shared evidentiary basis for accountability in which disability-related realities are (i) **represented** in audit-relevant records (e.g., accessibility barriers, reasonable accommodations, procedural burdens), (ii) **rendered interpretable** through explicit definitions, metadata, and decision rules, and (iii) **made traceable and contestable** through documentation standards and accessible grievance pathways that enable correction of misclassification and omission. Under this account, mistrust often emerges when experiential judgments of service adequacy and dignity are not legible within administrative records, while officials’ compliance and performance judgments rely on data that systematically understate exclusion. Strengthening representation, traceability, and contestability enables auditability for public organizations while improving recognizability and challenge ability for service users—thereby reducing misinterpretation and supporting comparable understanding of S, A, and C across stakeholder groups over time. Third, it identifies a research agenda for disability-inclusive digital government that connects information governance, audit institutions, and public administration paradigms to the practical design of trustworthy and equitable public services.

## Conceptual foundations

This article develops an integrated account of disability-inclusive accountability in digital-era public services by organizing the review through three interlocking lenses: public service delivery for people with disabilities (S), audit data governance (A), and data-driven culture (C). These lenses provide an analytic “grammar” for linking inclusion, accountability, and trust without presuming that different stakeholder groups rely on fundamentally different constructs. The systematic literature review (SLR) then enables us to specify the component structure of S and A and to synthesize directional relationships among S–A–C; these synthesis outputs are reported in the Results section.

### Service delivery for people with disabilities (S)

Inclusive public policy is grounded in the normative claim that public institutions must enable all people—especially those facing structural disadvantage—to access rights, opportunities, and public value on an equal basis. In disability-related public services, inclusion is not reducible to the existence of a programme; it depends on whether administrative rules, procedures, and information infrastructures are designed to recognize diverse needs and remove barriers to participation. The Convention on the Rights of Persons with Disabilities (CRPD) codifies state obligations to ensure accessibility, non-discrimination, and effective participation across domains of public life [5].

Disability scholarship and global evidence commonly conceptualize disability as arising from the interaction between impairments and social, environmental, and institutional barriers. For public service delivery, this implies that exclusion often emerges upstream—through forms, evidentiary requirements, verification rules, and inaccessible communication channels—rather than only at frontline encounters [18]. Digitalization can compound these risks when platforms, identity/eligibility checks, or data standards are not designed for accessibility and diverse support needs, making information infrastructures a core determinant of inclusion rather than a neutral technical backdrop [5].

A disability justice perspective further sharpens what counts as “inclusive” by foregrounding intersectionality, collective access, and the lived experiences of those most marginalized within disability communities. This lens is directly relevant to administrative information practices because it asks whose knowledge counts, who bears harms from misclassification or invisibility, and how administrative systems distribute burdens and benefits. In disability-related services, data omissions, rigid categorization, and misrepresentation are therefore justice issues—not merely technical quality issues [19]. From an accountability standpoint, inclusive policy thus implies **inclusive evidence**: credible performance claims require information that is accessible, traceable, and meaningfully representative of disabled people’s service realities.

### Audit data governance (A)

Public-sector data governance is widely understood as an institutional capability that assigns decision rights and accountabilities over data and operationalizes them through roles, standards, metadata, quality controls, access rules, and interoperability arrangements. In public administration, these mechanisms translate public values—legality, transparency, and procedural fairness—into actionable decisions about what data are collected, how they are defined, which documentation accompanies them, how they can be shared across agencies, and what constitutes legitimate evidence for performance and oversight [13].

This review narrows the governance object to audit-relevant data: the records, classifications, and documentation through which public organizations render service performance and administrative decisions auditable. Audit and performance systems are not neutral mirrors of reality; they privilege particular forms of evidence, stabilize what counts as “good performance,” and can crowd out experiential forms of knowing [6]. The implications are especially salient in disability-related services where administrative categories often mediate access to rights and support. When eligibility rules, evidentiary thresholds, and administrative classifications fail to capture relevant barriers—or when service encounters are recorded in ways that systematically understate exclusion—organizations may appear compliant while producing inequitable outcomes.

Public administration paradigms help explain why audit-relevant data become consequential and how accountability logics shape what is made legible as evidence. Efficiency-oriented managerialism associated with New Public Management (NPM) prioritizes measurable results and standardized indicators [8], which can intensify “audit society” dynamics and flatten lived realities when what is measurable becomes what matters [6]. New Public Service (NPS) emphasizes democratic citizenship and public value, implying that administrative information used to justify action should be intelligible and contestable to those affected [7]. New Public Governance (NPG) highlights networked delivery and relational accountability, elevating the importance of transparency, traceability, and coordination across organizational boundaries—conditions that depend on interoperable, well-documented information practices [20]. Taken together, these paradigms motivate a governance lens that is simultaneously auditable (for institutional accountability) and inclusion-sensitive (for disability equity and trust).

To specify what “good” governance should protect and enable, the review draws on rights-, equity-, and trust-oriented governance principles that treat data governance as a rights-bearing public capability rather than a purely managerial control function. Transform Health’s Health Data Governance Principles foreground safeguards against harm, responsible use for public value, and the conditions under which benefits and burdens of data-driven systems are distributed equitably [14]. These principles provide a normative and operational anchor for A: governing audit-relevant data must protect people and communities from harm, enable accountable value creation through data use, and ensure that data practices do not systematically disadvantage those already facing structural barriers.

Audit scholarship and practice further clarify why governing audit-relevant data is central to democratic accountability and public trust. INTOSAI’s public value framing positions Supreme Audit Institutions (SAIs) as actors expected to strengthen transparency and accountability and to “make a difference” to citizens’ lives [12]. IDI’s strategic management guidance makes the trust relationship explicit by linking internal capability improvements to societal outcomes, including enhanced democracy and public trust [11]. This relationship is central to our argument. Where audit-relevant data lack traceability, clear definitions, documented decision rules, or consistent classifications, even technically sound audits may have difficulty evidencing relevance and credibility in ways that maintain public trust [6,11].

### Data-driven culture (C)

Governance designs are enacted—or undermined—through everyday organizational routines. A data-driven organization is sustained less by tools than by culture: shared norms about what counts as evidence, how uncertainty is handled, and who can question decisions. Anderson defines data-driven culture as a set of reinforcing commitments that allow data to improve decisions at scale, including openness and trust, broad data literacy, goals-first reasoning, inquisitiveness and challenge, iterative learning, and resistance to authority-based decision-making when evidence contradicts senior opinion [15]. These cultural commitments are directly consequential for audit-relevant data because they shape whether staff treat data quality and documentation as collective responsibilities, whether decision rules are applied and recorded consistently, and whether errors and misclassifications are surfaced and corrected rather than normalized through workarounds.

More broadly, organizational change research suggests that reforms become durable only when leadership, incentives, and routines align so that new practices are embedded rather than symbolic [21,22]. In data-intensive public services, this implies that improvements in audit-relevant data governance depend not only on formal standards and controls but also on cultural capacity: valuing evidence integrity, accepting scrutiny, and learning from discrepancies between lived experience and administrative records [15].

### How S and C shape A

These lenses interlock in a mechanism-consistent way. **S** determines how service processes generate and structure audit-relevant data—what is recorded, what is omitted, and whether disability-related barriers and accommodations become legible as evidence. **A** determines whether those data are governed so they remain secure, interpretable, traceable, and contestable across organizational boundaries, enabling accountability that is both auditable and equity-affirming. **C** conditions whether these governance expectations operate as routine practice—through norms of documentation, challenge, correction, and learning—rather than remain aspirational.

Together, the conceptual foundations justify an integrated S–A–C account of disability-inclusive accountability and motivate the article’s synthesis focus: strengthening A is expected to improve the quality and legitimacy of accountability evidence by making audit-relevant data more representative of service realities, more traceable across service pathways, and more contestable when misclassification or omission occurs—while C enables these governance arrangements to function reliably in practice [5,6,8,14,15].

## Methodology

### Review approach and protocol

We conducted a protocol-guided systematic literature review (SLR) to consolidate evidence on how data governance mechanisms in public services enable or constrain auditability and the oversight use of audit-relevant data, and how these arrangements relate to trust, accountability, accessibility, and service equity in disability-relevant contexts. We report the review in line with PRISMA 2020, adapted to a conceptually oriented and methodologically heterogeneous corpus [23].

Because included records vary substantially in design, domains, constructs, and outcome reporting—and include empirical, conceptual, and standards-oriented contributions—we used a theory-led narrative synthesis [24]. Meta-analysis was not conducted due to heterogeneity in designs, constructs, and outcomes, and the inclusion of non-empirical sources.

To guide data extraction, coding, and the narrative synthesis, we organized evidence using three analytic lenses: service delivery for people with disabilities (S), audit data governance (A), and data-driven culture (C).

### Search strategy and study selection

#### Eligibility criteria and decision rules.

We included English-language records published between 2010 and 2025 that addressed audit-relevant data in public services and reported, theorized, or specified mechanisms that shape auditability, oversight use, accountability, or contestability of administrative decisions. Disability inclusion was operationalized using a decision rule: records were eligible if they (a) explicitly addressed disability, disabled people, accessibility, or reasonable accommodation in public services; or (b) examined public-service data governance/auditability arrangements with explicit equity- or accessibility-relevant implications for service access, eligibility determination, complaint/appeal pathways, or safeguards against exclusion. We excluded records that were not focused on disability/public services, were not relevant to audit/data governance, were editorials/commentaries, lacked sufficient methodological or conceptual detail to support extraction, or were duplicates/superseded records.

### Scope management

To avoid over-claiming disability specificity while still capturing governance mechanisms that shape inclusion in practice, we treated explicitly disability-focused records and more general public-service governance records (with stated implications for accessibility or service equity) as analytically distinct inputs into the narrative synthesis. This approach allowed us to retain a disability-inclusive focus while tracing how widely used administrative and digital-service infrastructures can enable or constrain inclusion through auditability-relevant mechanisms.

We searched Scopus as the primary bibliographic database because it provides broad interdisciplinary coverage across public administration, digital government, information systems, and social policy literatures and supports reproducible advanced query syntax. To reduce the risk that disability-relevant governance and accessibility evidence is under-indexed in bibliographic databases, we complemented Scopus retrieval with targeted searches of selected institutional and standards portals relevant to public-sector information policy, governance, assurance, and accessibility. The Scopus strategy used two complementary query variants (Search A and Search B), documented in full in Supporting Information S2 File, with database-side refine-values exports provided in Supporting Information S3 File (Search A) and S4 File (Search B). Targeted-source searching was executed using predefined source lists and keyword variants specified in the protocol (S1 File) and search documentation (S2 File), and all records—regardless of source—were screened using the same eligibility criteria and decision rules.

All records were imported into Zotero for screening; deduplication was performed and zero duplicates were removed (n = 0). No non-English records were removed prior to screening (n = 0). PRISMA stage counts and reasons for full-text exclusions are documented in Supplementary Material S2 Table (tabs ‘Review_Log’ and ‘Full-text_Exclusions’). Critical appraisal judgments are provided in the same workbook (Supplementary Material S2 Table, tab ‘Critical_Appraisal’). A consolidated evidence and coding matrix for the final corpus is provided in Supplementary Material S1 Table, and the completed PRISMA 2020 checklist is provided in Supplementary Material S1 Checklist. Supplementary Material S5 File provides the Zotero export of included records.

Critical appraisal (S2 Table) rated 11 records as high quality/low risk, 57 as moderate quality/some risk, and 57 as low quality/high or unclear risk. Synthesis emphasizes mechanisms supported by higher-quality evidence, while treating claims based primarily on lower-quality or weakly reported sources as tentative.

### Evidence matrix and coding (S–A–C lenses)

Data extraction and coding were recorded in one consolidated evidence and coding matrix (S1 Table). For each included record, we captured bibliographic information and extracted how the record treated audit-relevant data—including practices of collection, classification, linkage, access, transparency, and accountability—and what implications it discussed for trust, legitimacy, accessibility, and equity.

We then coded each record using an S–A–C scheme to maintain traceability from individual studies to the integrative framework:

- S codes capture service delivery arrangements and administrative procedures that shape access, user experience, and the generation of audit-relevant data.
- A codes capture governance mechanisms over audit-relevant data (roles, rules, controls, stewardship, assurance, transparency, and accountability structures).
- C codes capture organizational norms and capabilities that condition how audit-relevant data are interpreted, challenged, and acted upon.

Coding was iterative. We started with open coding during extraction, refined code definitions through repeated comparison across records, and consolidated overlapping labels. Records could receive multiple S/A/C codes when they contributed to more than one lens. The resulting coded matrix served as the analytic backbone for synthesis and for identifying recurring configurations across S–A–C.

### Critical appraisal: quality and risk of bias

To meet systematic review standards, we critically appraised the included records using tools appropriate to heterogeneous evidence types, and documented appraisal outcomes in Supplementary Material S2 Table (tab ‘Critical_Appraisal’).

Each record was first classified by evidence type (empirical study; conceptual/theoretical contribution; or standards/policy/grey literature).

Empirical studies were appraised using a Mixed Methods Appraisal Tool (MMAT)-inspired reporting-quality rubric (focused on clarity of aims, data and sampling description, and analytic transparency). Standards, policy, and grey literature were appraised using an Authority, Accuracy, Coverage, Objectivity, Date, and Significance (AACODS)-inspired rubric (authority, currency, coverage, accuracy, and objectivity). Conceptual/theoretical records were appraised for transparency of argument development, conceptual clarity, and engagement with prior scholarship.

We applied an explicit, cross-design rating rule to support consistent appraisal across heterogeneous evidence types. ‘High quality/low risk’ required clear alignment between the research question and design (or, for non-empirical records, clear purpose and scope), transparent data/materials and methods (or transparent sourcing and authority), coherent analytic logic, and substantiated claims with appropriate limitations. ‘Moderate quality/some risk’ reflected partial transparency or minor concerns (e.g., limited methodological detail, incomplete justification of analytic steps, or under-specified boundary conditions) that were unlikely to overturn the main contribution. ‘Low quality/clear risk’ reflected multiple concerns such as unclear sourcing or methods, weak linkage between evidence and claims, or insufficient detail to assess credibility. These ratings were used to weight interpretation: mechanisms supported by higher-quality evidence were treated as more robust, while patterns emerging mainly from lower-quality or weakly reported sources were framed as tentative propositions and agenda-setting signals rather than settled findings.

Where full texts were available, appraisal was based on full-text reading; where access constraints applied, appraisal relied on structured extraction from abstracts and source metadata. Uncertainty was explicitly recorded and treated as higher risk in interpretation.

Appraisal results were used to prioritize more transparent and methodologically robust evidence when characterizing mechanisms, and to frame lower-quality or weakly reported sources as tentative and agenda-setting. All appraisal uncertainties and decision-rule applications were recorded at the record level to preserve traceability and enable re-audit of the synthesis logic (see S2 Table and the linked extraction fields in S1 Table).

This approach supports reproducible synthesis in a methodologically diverse corpus where conventional intervention-focused risk-of-bias tools are not always applicable.

### Synthesis, robustness, and transparency

Synthesis proceeded through structured narrative comparison across records, focusing on (i) how audit-relevant data are produced and structured within public services, (ii) how governance arrangements condition the use of audit-relevant data for oversight and accountability, and (iii) how these dynamics relate to trust, accessibility, and equity in disability-relevant services [24]. Where primary studies reported quantitative associations, these were used descriptively to contextualize mechanisms; we did not pool effect sizes due to heterogeneity in designs, constructs, and outcomes.

To support robustness, we conducted sensitivity checks by (i) down-weighting records with limited methodological transparency, (ii) comparing patterns across digital-first versus hybrid/paper-mediated service contexts, and (iii) contrasting records with an explicit disability focus against those articulating general equity and accessibility principles applicable to disability-related services.

### Reporting bias and certainty assessment

Because the included corpus is methodologically heterogeneous and includes conceptual and standards-oriented sources, formal publication-bias tests (e.g., funnel-plot or small-study effects) were not appropriate. We therefore did not compute a meta-analytic certainty rating. Instead, we mitigated and transparently reported potential reporting biases through: (i) comprehensive database and targeted-source searching with full strategies provided (S2 File), (ii) dual-reviewer screening and data extraction with consensus procedures, and (iii) critical appraisal of each record with interpretation weighted by appraisal level (S2 Table). Accordingly, confidence in each synthesis claim is discussed in relation to the quality/risk-of-bias profile of the supporting evidence.

### Ethics statement

This study is a systematic literature review and narrative synthesis of publicly available sources and did not involve new data collection from human participants. Ethics approval and informed consent were therefore not required.

### Use of generative AI and AI-assisted tools

During manuscript preparation, the authors used ChatGPT to support language editing and formatting. The authors reviewed and revised all AI-assisted output and verified its consistency with the cited sources. The authors take full responsibility for the accuracy, validity, and integrity of the manuscript.

## Results

### Overview of the included corpus and analytic outputs

**Fig 1:**
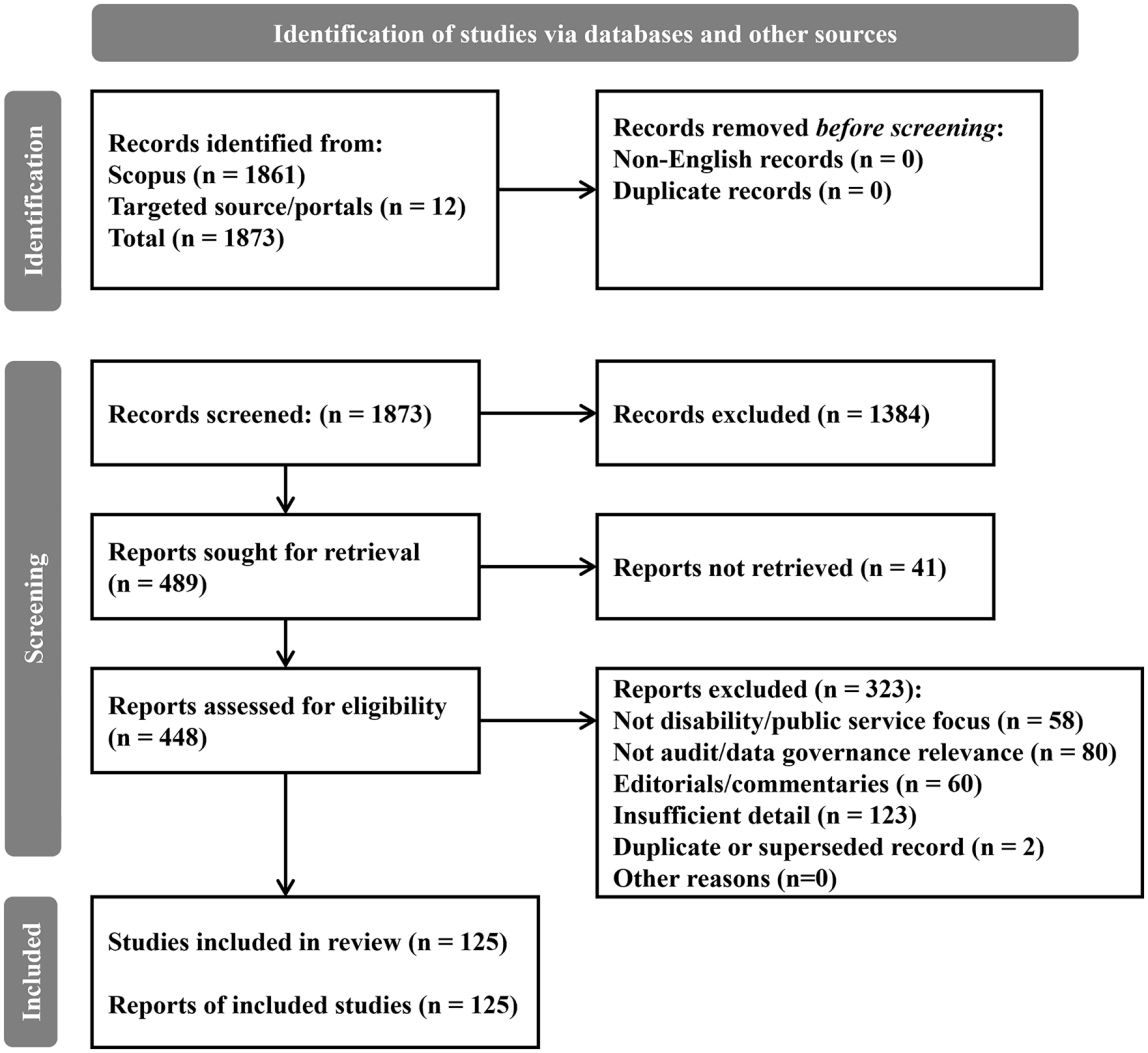
PRISMA 2020 flow diagram of the study selection process.

The systematic review yielded a final corpus of 125 English-language records synthesized through a theory-led narrative approach. The analytic output of the review is an integrated S–A–C account of how audit-relevant data is produced within public services, governed for oversight use, and interpreted and acted upon inside organizations. To preserve traceability, each record was coded and summarized in an evidence and coding matrix (S1 Table). The overall selection process is documented in the PRISMA 2020 flow diagram (Fig 1.) and the completed PRISMA 2020 checklist (S1 Checklist).

**Fig 2:**
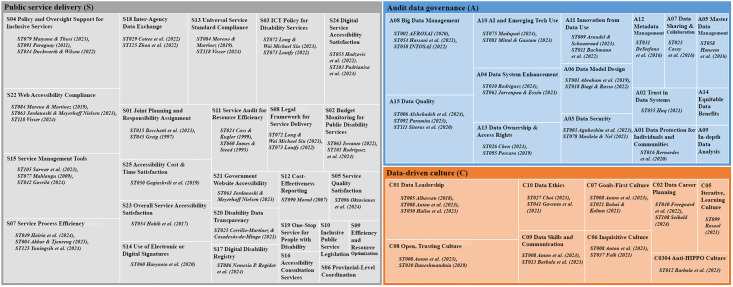
Hierarchy chart of prominent studies supporting the integrative S–A–C framework. The chart visualizes the most prominent studies identified in the review and their clustering across the three analytic lenses: service delivery for people with disabilities **(S)**, audit data governance **(A)**, and data-driven culture **(C)**.

To make the evidence structure more visible to readers, Fig 2 presents a hierarchy chart of the prominent studies underlying the integrative S–A–C framework.

Across the corpus, three recurrent findings structure the Results. First, service delivery arrangements shape whether audit-relevant data are generated in usable, comparable, and accessible forms for people with disabilities. Second, governance arrangements determine whether audit-relevant data can be used for accountability without producing new forms of harm or exclusion. Third, organizational culture conditions whether audit-relevant data are treated as contestable evidence—open to questioning and learning—or as a compliance artefact that reproduces institutional priorities.

Study selection results are summarized in Fig 1. In total, 1,861 records were identified from Scopus and 12 from targeted portals (total = 1,873). No records were removed as non-English (n = 0) or as duplicates (n = 0) prior to screening. Following title and abstract screening, 1,384 records were excluded and 489 reports were sought for retrieval; 41 reports could not be retrieved. We assessed 448 full-text reports for eligibility and excluded 323 reports with reasons (Not disability/public service focus, n = 58; Not audit/data governance relevance, n = 80; Editorials/commentaries, n = 60; Insufficient detail, n = 123; Duplicate or superseded record, n = 2; Other reasons, n = 0). The final corpus comprised 125 included records. Reasons and PRISMA stage counts are documented in Supplementary Material S2 Table (tabs ‘Full-text_Exclusions’ and ‘Review_Log’).

All screening and extraction steps were conducted using the written decision rules described above, and the resulting corpus, appraisal, and coding outputs are provided to enable independent verification (S1 and S2 Tables, S1 Checklist, S1–S5 Files).

### Quality and risk of bias appraisal across the included corpus

Supplementary Material S2 Table (tab ‘Critical_Appraisal’) reports the critical appraisal outcomes for all 125 included records: 11 were rated high quality/low risk, 57 moderate quality/some risk, and 57 low quality/high or unclear risk. Across evidence types, empirical records varied in reporting completeness, while standards/policy documents varied in authority and transparency of evidentiary basis.

These appraisal results were used to weight interpretation: mechanisms supported by higher-quality evidence are described as more robust, whereas mechanisms derived primarily from lower-quality or weakly reported sources are framed as tentative and used to define the research agenda.

### S lens: Service delivery arrangements that shape audit-relevant data

Across the service-delivery literature, the review shows that access barriers for people with disabilities are frequently produced by administrative processes and information practices—not only by “front-end” service design. In disability-relevant services, audit-relevant data are often created at points where users must prove eligibility, navigate classification, or repeatedly disclose sensitive information. These points of contact become consequential because they determine what the system records as “need,” “entitlement,” “non-compliance,” or “service failure,” and thus what later becomes visible (or invisible) to oversight.

#### S-1. Accessibility and information quality as preconditions for auditability.

Records emphasize that digital accessibility non-compliance can make service experiences systematically under-recorded or misrecorded, particularly when users cannot access official portals or forms through assistive technologies. When accessibility is treated as a legal/technical checklist rather than an operational requirement for service evidence, audit-relevant data become biased toward users who can comply with the dominant channel. This matters because audit-relevant data then describe institutional throughput rather than lived access. The corpus links this mechanism to the uneven observability of barriers and to weak feedback loops for correction [25,26].

#### S-2. Classification and eligibility processes as “data-generating bottlenecks”.

A recurring mechanism is that disability classification, eligibility screening, and documentation rules often function as bottlenecks that determine both service access and the content of audit-relevant data. Where classification categories are rigid, poorly contextualized, or not designed around diverse impairments, audit-relevant data can systematically under-represent unmet need and over-represent “procedural failure” by users. Conversely, service models that recognize heterogeneous impairments and allow flexible, user-responsive pathways tend to produce audit-relevant data that better represent accessibility constraints, grievances, and accommodation efforts [27,28].

#### S-3. Participation and local governance as sources of accountability evidence.

The corpus also indicates that local governance arrangements can create (or suppress) audit-relevant data that reflect user priorities. Where participation is embedded—through local consultative bodies, disability councils, or structured feedback mechanisms—service systems generate richer evidence about barriers, complaint handling, and “fit” between policy intent and service reality. This shifts audit-relevant data from being primarily transactional logs toward being evidence of responsiveness and inclusion [29].

#### S lens synthesis.

Taken together, S-coded evidence suggests that “auditability” in disability-relevant services is inseparable from the accessibility of service channels and the justice of classification and documentation practices. Service delivery determines not only *whether* audit-relevant data exist, but also *whose experiences* are legible to oversight.

### A lens: Governance mechanisms over audit-relevant data

A-coded records converge on a three-function account of audit data governance in public services. Across diverse domains, governance arrangements that improve oversight use of audit-relevant data tend to align with three functions: **protect people**, **promote value added**, and **prioritize equity**.

#### A-1. Protect people: safeguarding rights, privacy, and trust in audit-relevant data.

Across the corpus, governance is treated as inseparable from legitimacy. Records emphasize that audit-relevant data in disability-relevant contexts are frequently sensitive (health, impairment status, service dependency, location, and grievance narratives). Where governance is weak—unclear stewardship, porous access control, ambiguous sharing rules—oversight can become associated with surveillance and risk, reducing willingness to engage and lowering data quality. Conversely, arrangements framed as “public trust” duties, with strong protection, transparency about use, and meaningful accountability for misuse, are associated with stronger legitimacy conditions for oversight [30,31].

#### A-2. Promote value added: making audit-relevant data usable for oversight and learning.

A recurrent argument is that governance must enable audit-relevant data to support learning and improvement, not only compliance. This includes data quality management, metadata and provenance, interoperability across service systems, and the capacity to assemble coherent audit trails across organizational boundaries. In the auditing literature, this is closely linked to the feasibility of digital-era oversight approaches (e.g., analytics-enabled audits, risk sensing, and cross-source triangulation) that depend on the reliability and traceability of audit-relevant data [11,32–34].

#### A-3. Prioritize equity: ensuring audit-relevant data represent exclusion and enable remedy.

The equity function is prominent in disability-relevant records that critique “neutral” data systems as structurally exclusionary. Where disability is under-measured, misclassified, or treated as an afterthought, audit-relevant data cannot support equity-oriented accountability because exclusion is not observable as a governance problem. This theme is often framed as disability data justice: governance that ensures representation, community utility, and safeguards against data ableism and discrimination [28,35].

#### A lens synthesis.

Across the corpus, the strongest A-coded pattern is that oversight use of audit-relevant data is enabled when governance simultaneously (i) protects people, (ii) improves data usability and traceability, and (iii) makes exclusion measurable and contestable—rather than treating equity as an external normative add-on.

### C lens: Data-driven culture as the condition for meaningful oversight use

C-coded records consistently indicate that formal governance arrangements are insufficient if organizational culture discourages questioning, hides uncertainty, or treats audit-relevant data as a reputational risk rather than shared evidence. Across the corpus, data-driven culture is operationalized through leadership, ethics, openness, learning routines, and the social distribution of data literacy.

#### C-1. Data leadership and organizational enablement.

A recurring mechanism is that leadership shapes whether audit-relevant data are resourced and used as an organizational asset. Where leaders prioritize measurable outputs without equity-sensitive interpretation, audit-relevant data can support “performance theatre.” Where leaders instead treat data as a basis for learning and accountability—including scrutiny of unintended exclusion—audit-relevant data become actionable for service improvement [15,36].

#### C-2. Data ethics and the governance–culture interface.

Across records, ethics appears not as a standalone policy but as a cultural practice: how staff interpret privacy, consent, and proportionality in disability-relevant contexts. Ethical cultures reduce the risk that audit-relevant data are repurposed in ways that undermine trust (e.g., punitive profiling, deterrence effects, or “defensive” documentation practices). [15,36]

#### C-3. Open, questioning, and learning-oriented norms.

The corpus emphasizes that oversight value depends on whether staff can challenge data, surface uncertainty, and revise practices based on evidence. Cultures that reward questioning and iterative learning are more likely to use audit-relevant data to identify exclusionary processes and to sustain corrective action over time. [15]

#### C lens synthesis.

C-coded evidence positions culture as the mediator between having audit-relevant data and using them for inclusive accountability. In cultural environments dominated by hierarchy, blame avoidance, or anti-disclosure incentives, audit-relevant data are likely to be incomplete, strategically curated, or interpreted through institutional convenience.

### Cross-lens configurations: how S, A, and C co-produce inclusive (or exclusionary) accountability

Synthesizing across lenses, the review identifies recurring configurations that help explain why audit-relevant data sometimes strengthen accountability and equity, and sometimes reproduce exclusion.

#### Configuration 1: Accessible services + protective governance + learning culture → “inclusive auditability”.

When service channels are accessible and participation mechanisms generate user-relevant evidence (S), governance safeguards rights and ensures traceability (A), and culture supports questioning and learning (C), audit-relevant data can be used for oversight in ways that improve trust and reduce inequities. In these configurations, complaints, accommodation decisions, and service failures are legible to oversight and can trigger corrective action rather than being normalized as individual non-compliance. [15,29,31]

#### Configuration 2: Inaccessible services + weak governance + compliance culture → “exclusionary auditability”.

Where accessibility barriers push people with disabilities out of official channels (S), governance does not protect against misuse or bias (A), and culture prioritizes procedural compliance over learning (C), audit-relevant data tend to represent institutional throughput rather than access. Oversight in these contexts risks reinforcing exclusion because the system cannot “see” barriers as governance failures. [25,28,35]

#### Configuration 3: Strong technical governance without equity representation → “auditability without justice”.

A notable cross-lens finding is that improvements in data quality, metadata, and analytics capacity (A-2) do not guarantee equity if disability-relevant categories are missing, contested, or poorly contextualized (S-2) and if cultural norms discourage contestation of what counts as evidence (C-3). In such cases, audit-relevant data can become more technically auditable while remaining normatively blind to exclusion. [28,34,36]

#### Integrative S–A–C framework: pathways linking service delivery, audit data governance, and data-driven culture.

Synthesizing across the S–A–C lenses, we derive an integrative framework that explains how disability-inclusive service delivery (S), audit data governance (A), and data-driven culture (C) jointly shape the production, governance, interpretation, and use of audit-relevant data in public services. The framework specifies three linked pathways.

**Traceability from evidence to framework:** The integrative S–A–C framework is derived inductively from recurring, cross-record mechanisms identified through structured coding rather than imposed as an a priori model. Record-level traceability was maintained by linking each included record to specific S, A, and C codes in the evidence and coding matrix (S1), and by interpreting mechanisms in light of the critical appraisal ratings (S3). Accordingly, each pathway and configuration summarized below should be read as a mechanism-based synthesis that consolidates convergent patterns across the heterogeneous corpus, while indicating where claims are primarily supported by higher-quality empirical evidence versus conceptual, policy, or standards-oriented contributions.

**Fig 3:**
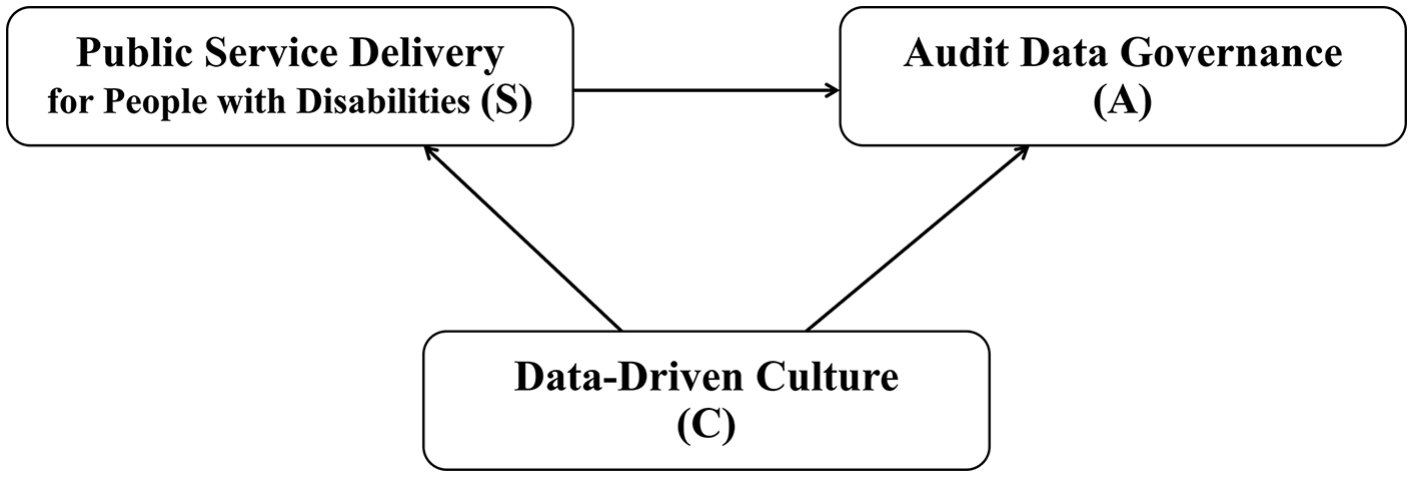
Integrative S–A–C framework showing three pathways (S → A, C → S, C → A) linking service delivery (S), audit data governance (A), and data-driven culture (C) in disability-inclusive public services.

Fig 3 presents the integrative S–A–C framework derived from the narrative synthesis.

An additional exploratory diagram is provided in Supporting information as S9 Fig.

### Pathway 1 (S → audit-relevant data): service delivery as the production site of audit-relevant data

Service delivery arrangements determine what is recorded, in what form, and whose experiences become legible to oversight. Accessibility of channels, classification and eligibility practices, and grievance/feedback routines shape whether audit-relevant data capture barriers and accommodations—or instead primarily reflect throughput and procedural completion [25,27].

### Pathway 2 (A → oversight use): governance as the enabling condition for legitimate, usable, and equity-sensitive oversight

Audit data governance conditions whether audit-relevant data can be accessed, trusted, and used for accountability without undermining rights and trust. Across the corpus, governance mechanisms cluster around (i) protecting people (privacy, security, legitimacy), (ii) promoting value added (quality, provenance, interoperability, traceability), and (iii) prioritizing equity (representation and contestability of exclusion in data) [28,33,35,37,38].

### Pathway 3 (C as a conditioning mechanism): culture shapes interpretation, challenge, and learning from audit-relevant data

Data-driven culture influences whether audit-relevant data are treated as contestable evidence that can trigger learning and corrective action, or as compliance artefacts that reinforce institutional priorities. Leadership, ethics, openness to challenge, and organizational learning routines emerge as central cultural mechanisms shaping how audit-relevant data translate into action [15].

Cross-lens interaction (S × A × C): inclusive auditability versus exclusionary auditability. The framework therefore predicts that inclusive accountability is most plausible when accessible service processes generate equity-relevant audit-relevant data (S), governance enables transparent and contestable use with rights protections (A), and culture supports questioning and learning rather than blame avoidance (C). Conversely, “exclusionary auditability” emerges when inaccessible or burdensome service processes produce biased traces (S), governance limits contestability or obscures provenance (A), and culture privileges easily measured outputs over equity-sensitive interpretation (C) [7,39,40].

### Positioning notes

This integrative S–A–C framework is derived from narrative synthesis rather than validated measurement. It therefore provides a basis for propositions and subsequent empirical testing (e.g., measurement development via EFA/CFA and structural model testing), rather than constituting a tested causal model within the present review.

### Robustness checks across record types and contexts

The S–A–C patterns above remained substantively stable when considering differences in (i) digital-first versus hybrid/paper-mediated service contexts, and (ii) records with explicit disability focus versus those addressing accessibility and equity principles with clear applicability to disability-relevant services. The main variation concerned *where* exclusion manifested: in digital-first settings, accessibility and data linkage were more salient; in hybrid settings, documentation burden and discretionary gatekeeping were more prominent. Across both, however, the same cross-lens logic held: service design shapes the evidentiary record; governance shapes legitimate use; culture shapes meaningful interpretation and action.

## Discussion

### What this review adds

This systematic review advances an integrative account of how audit-relevant data in public services become consequential for disability-inclusive accountability. Rather than treating auditability as a technical property of datasets or systems, the synthesis shows that audit-relevant data are co-produced through (S) service delivery arrangements, (A) audit data governance mechanisms, and (C) data-driven culture. The main contribution is therefore a framework that explains why oversight can appear robust—through compliance metrics, dashboards, or formal audit trails—while still failing to detect exclusionary barriers experienced by people with disabilities.

Given the methodological and conceptual heterogeneity of the included records, the S–A–C framework is presented as a mechanism-based synthesis rather than causal proof. The pathways and configurations therefore articulate plausible explanatory mechanisms and boundary conditions that can be tested in future empirical designs (e.g., comparative case studies, audits of administrative datasets, or mixed-method evaluations), rather than estimating pooled effect sizes or asserting universal causal relations.

Interpreting findings in light of study quality and risk of bias is central to the claims a systematic review can make. Accordingly, we treated the appraisal outputs (Supplementary Material S2 Table) as part of the evidentiary base: conclusions emphasize mechanisms consistently supported by higher-quality/low risk-of-bias empirical studies and by transparent, authoritative standards or policy sources, while areas dominated by lower-quality evidence are presented as priorities for primary research and evaluation.

### Interpreting the results through the S–A–C framework

#### S: service delivery determines whose experiences are legible to oversight.

Across the corpus, service processes are not neutral conduits; they determine which events become recorded as audit-relevant data and which barriers remain invisible. In disability-relevant settings, accessibility of digital channels, documentation burden, and classification/eligibility regimes shape whether the administrative record captures accommodations and barriers, or whether it primarily records procedural completion. This aligns with a rights-based view of disability policy monitoring in which data and audit practices are meaningful only when they support equality and inclusive access in practice [27]. It also clarifies why accessibility compliance in e-government is not merely a user-experience issue but an accountability prerequisite: inaccessible channels systematically bias audit-relevant data toward those who can comply with dominant service pathways [25].

#### A: governance determines whether audit-relevant data can support legitimate and equity-sensitive oversight.

The review identifies governance as the enabling condition for oversight use. Consistent with structured reviews of data governance, governance mechanisms shape the allocation of roles, access rights, controls, stewardship, and accountability arrangements that determine data quality and interpretability [37,38]. In the disability-relevant corpus, the governance question is not only “can we audit?” but also “can we audit without harm, and in ways that surface exclusion?” This is where disability data justice becomes essential: when disability is under-represented, misclassified, or treated as an afterthought, audit-relevant data cannot support equity-oriented accountability because inequity is not observable as a governance failure [28,35]. The auditing-oriented literature further reinforces that analytics-enabled oversight depends on traceability, provenance, and contextual completeness—properties that are governance achievements, not inherent features of technology [33].

#### C: culture conditions whether oversight becomes learning rather than compliance theatre.

Culture mediates the relationship between having audit-relevant data and using them to improve equity. Data-driven cultures that value questioning, ethical reflection, and learning loops are more likely to treat anomalies (e.g., repeated drop-offs, high appeal rates, systematic non-take-up) as signals requiring investigation and redesign. By contrast, cultures that prioritise easily countable outputs risk converting audit-relevant data into “performance accounts” that legitimate exclusionary efficiency. This cultural mechanism resonates with practical accounts of building data-driven organisations, where leadership, ethics, openness, and learning routines are decisive for making data actionable and contestable [15].

### Theoretical implications

#### The review contributes in three ways.

First, it bridges digital government and auditing by reframing auditability as a socio-technical outcome. Accountability in digital systems is shaped by what becomes observable and contestable through data, echoing concerns about accountability in algorithmic and data-intensive decision-making [39].

Second, it extends data governance debates by integrating equity as a core governance function rather than an external normative add-on. Data justice scholarship argues that fairness and accountability require attention to how data infrastructures distribute visibility, burden, and harm [40]. The S–A–C framework specifies how this justice concerns materialise operationally in public services.

Third, it strengthens public administration interpretations of inclusion by showing that user participation and responsiveness are not merely democratic ideals but also mechanisms that improve the evidentiary basis for oversight. Classic participation arguments and the New Public Service perspective support the view that accountability depends on meaningful engagement and responsiveness to citizens lived realities, including marginalised groups [7,9,41].

#### Practical implications.

The synthesis implies that improving oversight cannot be achieved by governance reforms alone if service channels remain inaccessible and organisational cultures discourage contestation.

**Implication 1:** treat accessibility as an accountability control, not a compliance afterthought (S). Accessibility gaps bias the administrative record and weaken oversight. Agencies should therefore govern accessibility as part of auditability: ensuring accessible service channels, documenting accommodations, and capturing barrier-related events as part of the audit-relevant record [25].

**Implication 2:** calibrate audit data governance to protect, add value, and prioritise equity (A). Governance should explicitly define stewardship over audit-relevant data, purpose limitation for sensitive disability-related information, tiered access for legitimate scrutiny, and mechanisms that enable correction and contestation. Data governance frameworks already emphasise roles, controls, and quality; the disability-relevant literature adds that representation and contestability are essential for equity-sensitive oversight [28,35,37].

**Implication 3:** design cultural routines that operationalise learning from audit-relevant data (C). Oversight becomes meaningful when anomalies trigger inquiry and redesign rather than blame avoidance. Agencies should institutionalise learning loops (e.g., routine review of appeals, drop-offs, accommodation requests, and complaint narratives), and cultivate norms that enable staff to question metrics and challenge “official accounts” when they conflict with user experience [15].

### Implications for future research and measurement development

Because this article reports a synthesis rather than a validated causal model, the framework implies a clear empirical agenda.

#### Proposition set (for subsequent empirical testing).

**P1 (S → A):** More accessible and user-responsive service delivery processes are associated with stronger audit data governance performance (via clearer, more complete, and more contestable audit-relevant data).

**P2 (A → accountability outcomes):** Stronger audit data governance (protect–value–equity) is associated with higher perceived legitimacy and trust in oversight, mediated by the transparency and contestability of audit-relevant data.

**P3 (C as moderator):** Data-driven culture strengthens the positive association between audit data governance and equity-sensitive oversight use by enabling questioning, ethical reflection, and learning routines.

**P4 (S × A × C configuration effect):** Inclusive auditability is most likely under the joint configuration of accessible service delivery (S), calibrated governance (A), and learning-oriented culture (C); exclusionary auditability is most likely when one or more elements are weak.

**Measurement agenda:** Future work can operationalise S, A, and C as latent constructs, develop indicators grounded in the coded evidence matrix, and validate measurement using EFA/CFA prior to structural model testing. In disability-relevant contexts, measurement development should incorporate representation and contestability—so that equity is not reduced to a residual outcome but embedded within what is measurable and actionable [28,40].

### Limitations

Several limitations should be considered when interpreting these findings. First, although the search strategy was designed for transparency and reproducibility, Scopus was used as the primary bibliographic database and the review was limited to English-language records. Eligible studies indexed exclusively in other databases or published in other languages may therefore have been missed. We mitigated this risk by conducting targeted searches of selected institutional and standards portals relevant to public-sector information policy, governance, assurance, and accessibility, and by applying the same eligibility criteria across all sources; nevertheless, the synthesis should be interpreted as a structured consolidation of recurring mechanisms rather than an exhaustive census of all relevant publications.

Second, the corpus spans empirical studies, conceptual/theoretical contributions, and standards- or policy-oriented records with diverse designs and outcome reporting. This heterogeneity supports the review’s mechanism-focused narrative synthesis but limits direct comparability across records and precludes meta-analysis or conventional statistical assessments of reporting bias. Our conclusions therefore emphasize patterns that recur across multiple records and that remain coherent when weighted by critical appraisal, while treating weaker or single-source patterns as propositions requiring further testing.

Third, disability inclusion was addressed through an explicit decision rule that combined disability-specific evidence with more general public-service governance evidence where accessibility- or equity-relevant implications were stated. This approach strengthens coverage of governance mechanisms that shape inclusion in practice, but it also requires careful interpretation: not all included records are disability-specific, and the transferability of general governance mechanisms to disability-inclusive contexts may vary by service domain, legal safeguards, and institutional arrangements. We therefore state boundary conditions and avoid over-claiming disability specificity where the underlying evidence is primarily generic.

## Conclusion

Overall, the review indicates that audit-relevant data can strengthen disability-inclusive accountability only when service delivery generates equity-relevant evidence (S), audit data governance enables legitimate and contestable oversight (A), and data-driven culture sustains learning and ethical judgement (C). The integrative S–A–C framework provides a coherent explanation for why digital-era oversight can simultaneously expand audit capacity and reproduce exclusion—and it defines a concrete agenda for subsequent measurement development and empirical testing.

## Supporting information

S1 Table Evidence and coding matrix for included records (n = 125). Study-level extraction and coding used for the narrative synthesis, including bibliographic details and coded evidence aligned with the S–A–C analytic framework‌‌.
(XLSX)

S2 Table Critical appraisal (quality and risk of bias) summary for included records (n = 125). Study-level critical appraisal outcomes reported in a format appropriate to the heterogeneous evidence types included in the review.
(XLSX)

S1 Checklist PRISMA 2020 checklist. Completed PRISMA 2020 checklist indicating where each reporting item is addressed in the manuscript and supplementary materials.
(DOCX)

S1 File Review protocol. Protocol specifying the review questions, eligibility criteria, information sources, screening and extraction procedures, analytic approach, and any deviations from planned methods.
(DOCX)

S2 File Full search strategies for each information source (Scopus and targeted sources). Verbatim search strings, limits/filters (including language restrictions), and search dates for Scopus, plus the targeted-source search approach used to identify additional records.
(DOCX)

S3 File Scopus search output (Search A) summary (refine values). Exported Scopus “refine values” summary for Search A used to document retrieval counts and filtering (including the English-language restriction).
(XLSX)

S4 File Scopus search output (Search B) summary (refine values). Exported Scopus “refine values” summary for Search B used to document retrieval counts and filtering (including the English-language restriction).
(XLSX)

S5 File Zotero library export for included records (n = 125). Exported Zotero library file for the final included set (n = 125), provided to support traceability of the included corpus and reference management.
(XLSX)

S1 Fig Illustrative example of cross-lens relationships across service delivery, audit data governance, and data-driven culture. This supplementary figure is provided as an illustrative example to show how relationships may be represented across the three analytic lenses used in this review: service delivery (S), audit data governance (A), and data-driven culture (C). It is intended to support conceptual interpretation of the integrative S–A–C framework and should not be read as a standalone synthesis result.
(TIF)
