# Correction: Audit data governance for disability-inclusive public services: A systematic review and integrative S–A–C framework

**DOI:** 10.1371/journal.pone.0358691

**Published:** 2026-09-16

**Authors:** Sitthisak Chaiyasuk, Krish Rugchatjaroen, Somboon Sirisunhirun, Nopraenue Sajjarax Dhirathiti, Somsak Amornsiriphong, Phut Ploywan

All authors were incorrectly listed as contributing equally to this work. The correct equal contribution notes are as follows: Sitthisak Chaiyasuk and Krish Rugchatjaroen contributed equally to this work. Somboon Sirisunhirun, Nopraenue Sajjarax Dhirathiti, Somsak Amornsiriphong, and Phut Ploywan also contributed equally to this work.
