# Supplementary material for: Audit data governance for disability-inclusive public services: A systematic review and integrative S–A–C framework
Source: PLoS One. 2026 May 22;21(5):e0350135. doi: 10.1371/journal.pone.0350135 (PMC13196965; doi:10.1371/journal.pone.0350135)
Supplement: S1 Checklist — Completed PRISMA 2020 checklist indicating where each reporting item is addressed in the manuscript and supplementary materials. (DOCX) [file pone.0350135.s003.docx]

| **Section and Topic** | **Item #** | **Checklist item** | **Location where item is reported** |
| --- | --- | --- | --- |
| **TITLE** | | |  |
| Title | 1 | Identify the report as a systematic review. | Title page (Title) and Abstract (first sentence identifying the work as a systematic review). |
| **ABSTRACT** | | |  |
| Abstract | 2 | See the PRISMA 2020 for Abstracts checklist. | ABSTRACT section. |
| **INTRODUCTION** | | |  |
| Rationale | 3 | Describe the rationale for the review in the context of existing knowledge. | INTRODUCTION section (rationale/context). |
| Objectives | 4 | Provide an explicit statement of the objective(s) or question(s) the review addresses. | INTRODUCTION section (objectives/research focus). |
| **METHODS** | | |  |
| Eligibility criteria | 5 | Specify the inclusion and exclusion criteria for the review and how studies were grouped for the syntheses. | METHODOLOGY section, under 'Search strategy and study selection' (eligibility criteria). |
| Information sources | 6 | Specify all databases, registers, websites, organisations, reference lists and other sources searched or consulted to identify studies. Specify the date when each source was last searched or consulted. | METHODOLOGY section, under 'Search strategy and study selection' (information sources). |
| Search strategy | 7 | Present the full search strategies for all databases, registers and websites, including any filters and limits used. | Supporting Information S5 (Full search strategies). |
| Selection process | 8 | Specify the methods used to decide whether a study met the inclusion criteria of the review, including how many reviewers screened each record and each report retrieved, whether they worked independently, and if applicable, details of automation tools used in the process. | METHODOLOGY section, under 'Search strategy and study selection' (selection process and reviewer checks). |
| Data collection process | 9 | Specify the methods used to collect data from reports, including how many reviewers collected data from each report, whether they worked independently, any processes for obtaining or confirming data from study investigators, and if applicable, details of automation tools used in the process. | METHODOLOGY section, under 'Data extraction and coding'; record-level summaries in S1 Table (evidence and coding matrix). |
| Data items | 10a | List and define all outcomes for which data were sought. Specify whether all results that were compatible with each outcome domain in each study were sought (e.g. for all measures, time points, analyses), and if not, the methods used to decide which results to collect. | METHODOLOGY section, under 'Data extraction and coding'; extraction fields and coding recorded in S1 Table (evidence and coding matrix). |
|  | 10b | List and define all other variables for which data were sought (e.g. participant and intervention characteristics, funding sources). Describe any assumptions made about any missing or unclear information. | METHODOLOGY section, under 'Synthesis, robustness, and transparency' (analytic focus and synthesis outputs). |
| Study risk of bias assessment | 11 | Specify the methods used to assess risk of bias in the included studies, including details of the tool(s) used, how many reviewers assessed each study and whether they worked independently, and if applicable, details of automation tools used in the process. | METHODOLOGY section: 'Critical appraisal: quality and risk of bias'; Supporting Information S3. |
| Effect measures | 12 | Specify for each outcome the effect measure(s) (e.g. risk ratio, mean difference) used in the synthesis or presentation of results. | N/A (no pooled effect measures; theory-led narrative synthesis described under 'Synthesis, robustness, and transparency'). |
| Synthesis methods | 13a | Describe the processes used to decide which studies were eligible for each synthesis (e.g. tabulating the study intervention characteristics and comparing against the planned groups for each synthesis (item #5)). | METHODOLOGY section, under 'Synthesis, robustness, and transparency' (process for deciding study eligibility for each synthesis; S–A–C coding frame). |
|  | 13b | Describe any methods required to prepare the data for presentation or synthesis, such as handling of missing summary statistics, or data conversions. | METHODOLOGY section, under 'Synthesis, robustness, and transparency' (data preparation/standardisation for synthesis). |
|  | 13c | Describe any methods used to tabulate or visually display results of individual studies and syntheses. | METHODOLOGY section, under 'Synthesis, robustness, and transparency' (methods to tabulate/visualise results; evidence matrix in S1 Table). |
|  | 13d | Describe any methods used to synthesize results and provide a rationale for the choice(s). If meta-analysis was performed, describe the model(s), method(s) to identify the presence and extent of statistical heterogeneity, and software package(s) used. | METHODOLOGY section, under 'Synthesis, robustness, and transparency' (methods to synthesise results; theory-led narrative synthesis). |
|  | 13e | Describe any methods used to explore possible causes of heterogeneity among study results (e.g. subgroup analysis, meta-regression). | METHODOLOGY section, under 'Synthesis, robustness, and transparency' (exploration of heterogeneity/configurations within S–A–C). |
|  | 13f | Describe any sensitivity analyses conducted to assess robustness of the synthesized results. | METHODOLOGY section, under 'Synthesis, robustness, and transparency' (sensitivity checks/robustness procedures). |
| Reporting bias assessment | 14 | Describe any methods used to assess risk of bias due to missing results in a synthesis (arising from reporting biases). | Not assessed; recommended: state under 'Synthesis, robustness, and transparency' that reporting-bias assessment was not feasible for this heterogeneous corpus. |
| Certainty assessment | 15 | Describe any methods used to assess certainty (or confidence) in the body of evidence for an outcome. | METHODOLOGY section: 'Reporting bias and certainty' (formal certainty assessment not undertaken; qualitative confidence communicated via appraisal). |
| **RESULTS** | | |  |
| Study selection | 16a | Describe the results of the search and selection process, from the number of records identified in the search to the number of studies included in the review, ideally using a flow diagram. | METHODOLOGY section, under 'Search strategy and study selection' (numbers) and Fig 1 (PRISMA 2020 flow diagram). |
|  | 16b | Cite studies that might appear to meet the inclusion criteria, but which were excluded, and explain why they were excluded. | Fig 1 (PRISMA 2020 flow diagram). |
| Study characteristics | 17 | Cite each included study and present its characteristics. | S1 Table (evidence and coding matrix; study/record characteristics) and RESULTS section (overview of included corpus). |
| Risk of bias in studies | 18 | Present assessments of risk of bias for each included study. | RESULTS section: 'Quality and risk of bias appraisal across the included corpus'; Supporting Information S3. |
| Results of individual studies | 19 | For all outcomes, present, for each study: (a) summary statistics for each group (where appropriate) and (b) an effect estimate and its precision (e.g. confidence/credible interval), ideally using structured tables or plots. | S1 Table (record-level summaries) and RESULTS section (narrative synthesis). |
| Results of syntheses | 20a | For each synthesis, briefly summarise the characteristics and risk of bias among contributing studies. | RESULTS section (synthesis of findings), including Fig 2 (hierarchy chart of prominent studies) and Fig 3 (integrative S–A–C framework). |
|  | 20b | Present results of all statistical syntheses conducted. If meta-analysis was done, present for each the summary estimate and its precision (e.g. confidence/credible interval) and measures of statistical heterogeneity. If comparing groups, describe the direction of the effect. | RESULTS section (mechanisms/configurations discussed across themes). |
|  | 20c | Present results of all investigations of possible causes of heterogeneity among study results. | METHODOLOGY section, under 'Synthesis, robustness, and transparency' (sensitivity/robustness), and DISCUSSION section (limitations/robustness). |
|  | 20d | Present results of all sensitivity analyses conducted to assess the robustness of the synthesized results. | N/A (no meta-analysis); narrative synthesis in RESULTS section. |
| Reporting biases | 21 | Present assessments of risk of bias due to missing results (arising from reporting biases) for each synthesis assessed. | METHODOLOGY section: 'Reporting bias and certainty assessment' (formal reporting-bias assessment not feasible; mitigation steps described). |
| Certainty of evidence | 22 | Present assessments of certainty (or confidence) in the body of evidence for each outcome assessed. | METHODOLOGY section: 'Reporting bias and certainty assessment' (no formal GRADE/meta-analytic certainty); DISCUSSION section: 'Limitations' (confidence linked to appraisal profile). |
| **DISCUSSION** | | |  |
| Discussion | 23a | Provide a general interpretation of the results in the context of other evidence. | DISCUSSION section (interpretation in context). |
|  | 23b | Discuss any limitations of the evidence included in the review. | DISCUSSION section (limitations of evidence and of review process). |
|  | 23c | Discuss any limitations of the review processes used. | DISCUSSION and CONCLUSION sections (implications for practice, policy, and future research). |
|  | 23d | Discuss implications of the results for practice, policy, and future research. | DISCUSSION section (equity/accessibility considerations where reported). |
| **OTHER INFORMATION** | | |  |
| Registration and protocol | 24a | Provide registration information for the review, including register name and registration number, or state that the review was not registered. | METHODOLOGY section, under 'Review approach and protocol' (the review was not registered); Supporting Information S4 File (review protocol). |
|  | 24b | Indicate where the review protocol can be accessed, or state that a protocol was not prepared. | METHODOLOGY section, under 'Review approach and protocol'; Supporting Information S4 (Review protocol). |
|  | 24c | Describe and explain any amendments to information provided at registration or in the protocol. | Supporting Information S4 File, 'Deviations from protocol' section (no amendments/deviations). |
| Support | 25 | Describe sources of financial or non-financial support for the review, and the role of the funders or sponsors in the review. | Funding section. |
| Competing interests | 26 | Declare any competing interests of review authors. | Competing interests section. |
| Availability of data, code and other materials | 27 | Report which of the following are publicly available and where they can be found: template data collection forms; data extracted from included studies; data used for all analyses; analytic code; any other materials used in the review. | Data Availability section; Supporting Information files S1–S8 (evidence/coding matrix, PRISMA checklist, critical appraisal table, review protocol, full search strategies, Scopus refine-values exports, and Zotero export). |

*From: Page MJ, McKenzie JE, Bossuyt PM, Boutron I, Hoffmann TC, Mulrow CD, et al.* ***The PRISMA 2020 statement: an updated guideline for reporting systematic reviews.*** *BMJ 2021;372:n71. doi:10.1136/bmj.n71.* ***This work is licensed under CC BY 4.0.***
