# Supplementary material for: Audit data governance for disability-inclusive public services: A systematic review and integrative S–A–C framework
Source: PLoS One. 2026 May 22;21(5):e0350135. doi: 10.1371/journal.pone.0350135 (PMC13196965; doi:10.1371/journal.pone.0350135)
Supplement: S1 File — Protocol specifying the review questions, eligibility criteria, information sources, screening and extraction procedures, analytic approach, and any deviations from planned methods. (DOCX) [file pone.0350135.s004.docx]

# S4 File. Review protocol

This protocol documents the a priori methods for the systematic review and provides sufficient detail to support reproducibility. Where methods deviated during conduct, deviations and rationale should be recorded in Section 9.

## 1. Review question and objectives

Primary question (define explicitly): How is audit-relevant data produced within public services, governed for oversight use, and interpreted and acted upon inside organizations, in disability-relevant contexts?

Objectives:

1) Identify and synthesize evidence on service delivery arrangements that shape audit-relevant data (S).

2) Identify and synthesize evidence on governance mechanisms over audit-relevant data (A).

3) Identify and synthesize evidence on data-driven cultures that condition interpretation and use of audit-relevant data (C).

4) Integrate S–A–C mechanisms into an explanatory framework for inclusive auditability.

## 2. Eligibility criteria

Inclusion criteria (pre-specify and keep consistent with the manuscript):

- Publication years: 2010–2025 (PUBYEAR > 2009 AND PUBYEAR < 2026). Note: records published before 2010 could still be included if identified through supplementary methods (e.g., citation searching) and meeting all inclusion criteria.
- Language: English.
- Document types: peer-reviewed empirical studies; mixed-methods studies; qualitative studies; structured conceptual analyses; relevant standards/policy/grey literature with sufficient transparency to support appraisal.
- Topical relevance: addresses at least one S–A–C lens in relation to auditability/oversight/accountability and disability-relevant service contexts (or explicitly generalizable accessibility/inclusion governance).

Exclusion criteria:

- Not focused on public services, governance, auditability/oversight, or disability-relevant inclusion/accessibility.
- Opinion pieces without methodological transparency or traceable sources (for non-empirical items).
- Duplicates; non-English; outside time window.

## 3. Information sources

Databases and sources: Scopus, complemented by targeted searches of selected institutional and standards portals. Date searched: 25 May 2025. Full Scopus search strings are provided in Supplementary Material S5; targeted sources and retrieval approach are specified in this protocol.

Targeted sources (pre-specify): institutional/standards portals relevant to public-sector information policy, accessibility, and governance (e.g., INTOSAI/IDI resources, major international policy and standards organizations).

## 4. Search strategy

Search strategies are documented in full in Supporting Information S5, including exact strings, filters, and the date each source was searched.

Key concepts and example term families (adapt to databases): audit/auditability/oversight/accountability; data governance/data quality/metadata/master data; public services/service delivery/administrative procedures; disability/accessibility/inclusion/rights; digital government/AI/automation.

## 5. Study selection

All records were imported into Zotero and de-duplicated. Deduplication was performed; zero duplicates were removed. Screening proceeded in two stages: (1) title/abstract screening and (2) full-text screening. Each stage was conducted independently by two reviewers, with disagreements resolved through discussion and, when required, adjudication by a third reviewer.

Stage 1: Title/abstract screening against eligibility criteria.

Stage 2: Full-text assessment for final inclusion.

Screening was performed by Reviewer A (Author 1) with Reviewer B (Author 2) independently screening a random subset (recommend ≥20%) or the full set; disagreements were resolved by consensus. Reasons for exclusion at full text were recorded and are summarized in Supplementary Material S3 (tab ‘Full-text_Exclusions’), with PRISMA stage counts documented in Supplementary Material S3 (tab ‘Review_Log’).

## 6. Data extraction

A structured extraction form was used to capture bibliographic details, setting/context, evidence type and design, relevant S–A–C mechanisms, and reported implications for trust, legitimacy, accessibility, and equity. Extraction outputs are compiled in S1 Table (evidence and coding matrix).

## 7. Critical appraisal (quality and risk of bias)

Empirical studies were appraised with a design-appropriate tool (recommendation for mixed-methods corpora: MMAT). Non-empirical sources (standards/policy/grey literature) were appraised using an authority-and-rigor checklist (recommendation: AACODS).

Quality appraisal was performed independently by six reviewers (the full author team) using design-appropriate tools and criteria (e.g., qualitative appraisal, observational-study appraisal, standards/policy relevance checks). Discrepancies were resolved by consensus, and overall ratings and brief justifications are reported in Supplementary Material S3 (tab ‘Critical_Appraisal’).

## 8. Synthesis approach

Given heterogeneity, synthesis followed a theory-led narrative approach structured by the S–A–C lenses. Mechanisms were compared across records and organized into cross-lens configurations; robustness checks incorporated appraisal outputs to distinguish robust versus tentative claims.

## 9. Deviations from protocol

No amendments or deviations from the protocolled eligibility criteria, screening workflow, or synthesis approach were made after commencement. Minor clarifications were made during manuscript preparation to strengthen cross-referencing between the protocol, PRISMA flow diagram, and supporting information. Scopus searches were restricted to English-language records at the query stage, as specified in the protocol.

**Reviewer team and decision process**
All six authors contributed to the review. Two reviewers conducted each screening stage independently, with third-reviewer adjudication for unresolved conflicts. Critical appraisal was completed independently by six reviewers. Final inclusion decisions were agreed by consensus and documented in Supplementary Material S3 (tabs ‘Review_Log’ and ‘Full-text_Exclusions’). Critical appraisal records are provided in Supplementary Material S3 (tab ‘Critical_Appraisal’).
