# Supplementary material for: Audit data governance for disability-inclusive public services: A systematic review and integrative S–A–C framework
Source: PLoS One. 2026 May 22;21(5):e0350135. doi: 10.1371/journal.pone.0350135 (PMC13196965; doi:10.1371/journal.pone.0350135)
Supplement: S2 File — Verbatim search strings, limits/filters (including language restrictions), and search dates for Scopus, plus the targeted-source search approach used to identify additional records. (DOCX) [file pone.0350135.s005.docx]

**S5 File. Full search strategies**

This file reports the complete search strings and key parameters for each information source. For each source, we report the date searched, platform/database, fields searched, limits applied, and the full query string(s) as executed.

**1. Scopus**

Date searched: 25 May, 2025.

**Fields:** TITLE-ABS-KEY. Limits: language=English; publication years 2010–2025 (PUBYEAR > 2009 AND PUBYEAR < 2026); no document-type restrictions at search stage.

**Search A (audit data governance in public services):**

TITLE-ABS-KEY( ("data governance" OR "information governance" OR "data stewardship" OR "data management" OR "data quality" OR metadata OR "data sharing" OR interoperability OR "data standard*" OR "data catalog*") AND (audit* OR auditability OR oversight OR "external audit" OR "supreme audit institution*" OR accountability) AND ("public service*" OR "public sector" OR government OR "public administration" OR "public organization*" OR "public agency" OR municipality OR "local government")) AND (LIMIT-TO(LANGUAGE, "English")) AND (PUBYEAR > 2009 AND PUBYEAR < 2026)

**Search B (disability/accessibility in public services with governance/audit lens):**

TITLE-ABS-KEY( (disabilit* OR "persons with disabilities" OR accessibility OR "reasonable accommodation" OR "inclusive service*" OR "universal design" OR "assistive technolog*") AND ("public service*" OR "public sector" OR government OR "public administration" OR "public agency" OR municipality OR "local government") AND ("data governance" OR "information governance" OR "data stewardship" OR "data management" OR "data quality" OR metadata OR audit* OR auditability OR oversight OR accountability)) AND (LIMIT-TO(LANGUAGE, "English")) AND (PUBYEAR > 2009 AND PUBYEAR < 2026)

**Results (Scopus documents):**

Search A = 641; Search B = 1,220; combined = 1,861 (English-language records).

**Evidence saved:**

Scopus refine-values exports saved as Scopus_exported_refine_values-A.xlsx (Search A) and Scopus_exported_refine_values-B.xlsx (Search B); included-record library export saved as zotero_125.xlsx (S8 File).

**2. Targeted institutional/standards sources**

Targeted sources were searched to capture standards, policy, and other grey literature not consistently indexed in Scopus. In the final corpus, targeted sources contributed 12 included records (see Table below).

| **Source / portal** | **Date searched** | **Search terms / navigation path** | **Records retrieved** | **Notes / rationale for inclusion** |
| --- | --- | --- | --- | --- |
| www.ssrn.com | 25 May 2025 | Site search for audit/data governance, accessibility, and public services; manual screening. | 3 | Contextual standards/policy scan; no unique additional records beyond database retrieval. Included records (n=3) derived from this source. |
| www.accessibilitychecker.org | 25 May 2025 | Site search for audit/data governance, accessibility, and public services; manual screening. | 1 | Contextual standards/policy scan; no unique additional records beyond database retrieval. Included records (n=1) derived from this source. |
| repository.nwu.ac.za | 25 May 2025 | Site search for audit/data governance, accessibility, and public services; manual screening. | 1 | Contextual standards/policy scan; no unique additional records beyond database retrieval. Included records (n=1) derived from this source. |
| www.audit.gov.cn | 25 May 2025 | Site search for audit/data governance, accessibility, and public services; manual screening. | 1 | Contextual standards/policy scan; no unique additional records beyond database retrieval. Included records (n=1) derived from this source. |
| aiforgood.itu.int | 25 May 2025 | Site search for audit/data governance, accessibility, and public services; manual screening. | 1 | Contextual standards/policy scan; no unique additional records beyond database retrieval. Included records (n=1) derived from this source. |
| cora.ucc.ie | 25 May 2025 | Site search for audit/data governance, accessibility, and public services; manual screening. | 1 | Contextual standards/policy scan; no unique additional records beyond database retrieval. Included records (n=1) derived from this source. |
| www.baltijapublishing.lv | 25 May 2025 | Site search for audit/data governance, accessibility, and public services; manual screening. | 1 | Contextual standards/policy scan; no unique additional records beyond database retrieval. Included records (n=1) derived from this source. |
| books.fupress.com | 25 May 2025 | Site search for audit/data governance, accessibility, and public services; manual screening. | 1 | Contextual standards/policy scan; no unique additional records beyond database retrieval. Included records (n=1) derived from this source. |
| www.apo-tokyo.org | 25 May 2025 | Site search for audit/data governance, accessibility, and public services; manual screening. | 1 | Contextual standards/policy scan; no unique additional records beyond database retrieval. Included records (n=1) derived from this source. |
| theodi.org | 25 May 2025 | Site search for audit/data governance, accessibility, and public services; manual screening. | 1 | Contextual standards/policy scan; no unique additional records beyond database retrieval. Included records (n=1) derived from this source. |
| heidiseibold.com | 25 May 2025 | Site search for audit/data governance, accessibility, and public services; manual screening. | 1 | Contextual standards/policy scan; no unique additional records beyond database retrieval. Included records (n=1) derived from this source. |
| www.oecd.org | 25 May 2025 | Site search for audit/data governance, accessibility, and public services; manual screening. | 1 | Contextual standards/policy scan; no unique additional records beyond database retrieval. Included records (n=1) derived from this source. |

PRISMA alignment note: The Scopus searches were executed with language=English limits applied at the query stage; therefore, no non-English records were removed prior to screening (non-English removed = 0). Search A returned 641 records and Search B returned 1,220 records; the merged Scopus dataset comprised 1,861 records after duplicate checking in Zotero (duplicates removed = 0).
