# Supplementary figures and images for: Audit data governance for disability-inclusive public services: A systematic review and integrative S–A–C framework
Source: PLoS One. 2026 May 22;21(5):e0350135. doi: 10.1371/journal.pone.0350135 (PMC13196965; doi:10.1371/journal.pone.0350135)

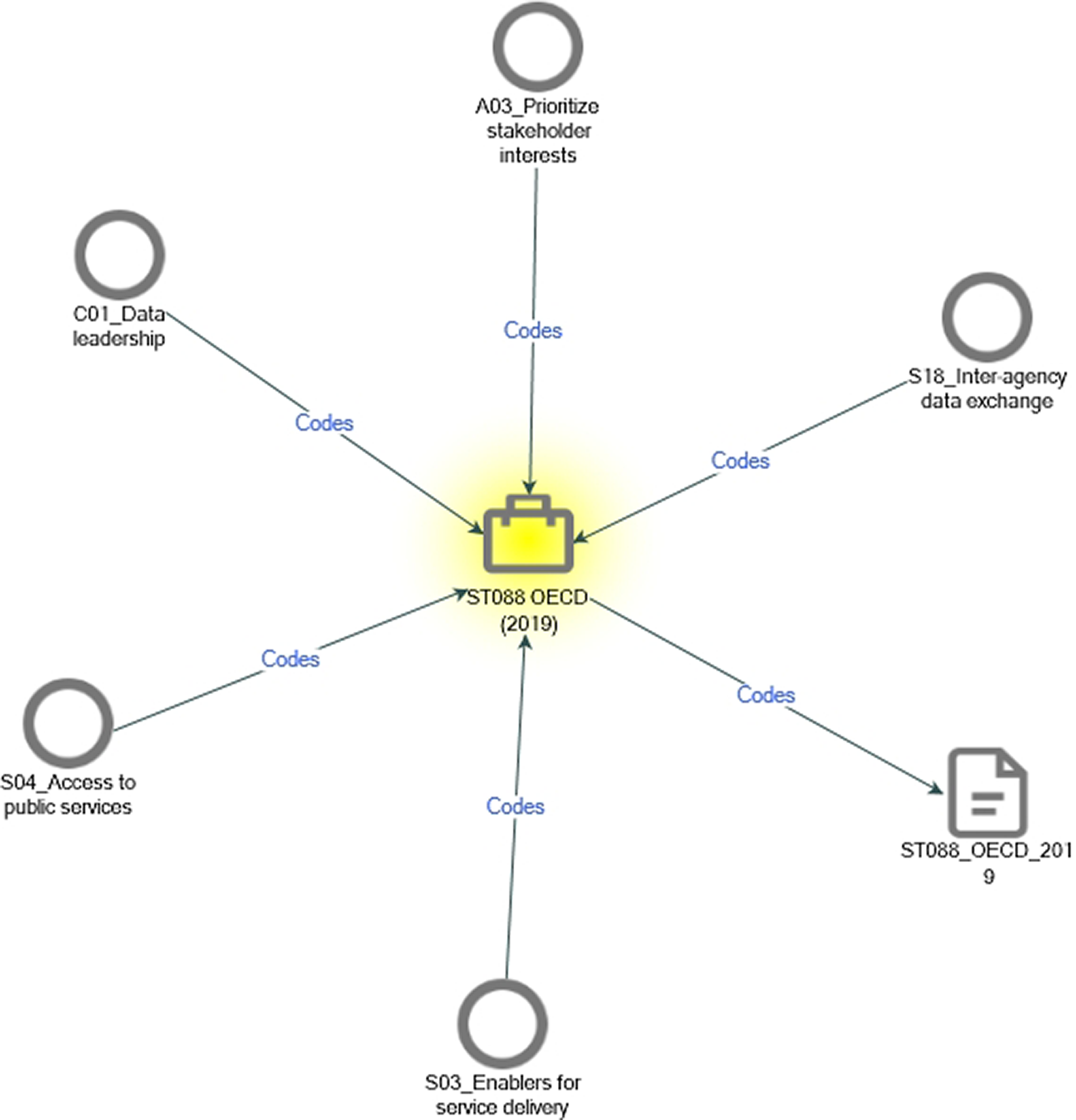

Supplement: S1 Fig — This supplementary figure is provided as an illustrative example to show how relationships may be represented across the three analytic lenses used in this review: service delivery (S), audit data governance (A), and data-driven culture (C). It is intended to support conceptual interpretation of the integrative S–A–C framework and should not be read as a standalone synthesis result. (TIF) [file pone.0350135.s009.tif]
